# Special Issue “Carbohydrates 2018”

**DOI:** 10.3390/ph13010005

**Published:** 2019-12-29

**Authors:** Amélia Pilar Rauter, Nuno Manuel Xavier

**Affiliations:** 1 Centro de Química e Bioquímica, Faculdade de Ciências, Universidade de Lisboa, Ed. C8, 5° Piso, Campo Grande, 1749-016 Lisboa, Portugal; nmxavier@fc.ul.pt; 2 Centro de Química Estrutural, Faculdade de Ciências, Universidade de Lisboa, Ed. C8, 5° Piso, Campo Grande, 1749-016 Lisboa, Portugal

This special issue of Pharmaceuticals has been dedicated to Carbohydrates on the occasion of the 29th International Carbohydrate Symposium, held at the Universidade de Lisboa from 15–19 July 2018. Recent findings and trends in carbohydrate science are presented and discussed in its nine articles. They are demonstrative of the relevance of carbohydrate research in medicinal chemistry and pharmaceutical sciences, and of the exciting opportunities that carbohydrate-based structures offer for the discovery of new solutions for therapy and diagnosis.

Carbohydrates are natural, multifunctional, and stereochemically rich molecules, playing important roles in biological processes relevant for health and disease. Embodying such structural features, these unique molecular entities can be transformed in a diversity of compounds applied as drugs, food supplements, as biologically active materials, in cosmetics, just to name a few of the wide uses of carbohydrates and their mimetics. Research in carbohydrates also covers a diversity of domains as highlighted in this special issue, containing contributions of experts in fields such as glycochemistry, molecular biology, computational chemistry, and materials science, that address the roles of carbohydrates to understand biological processes and to develop new approaches for disease diagnosis and treatment.

Kuttel and Ravenscroft describe a molecular modeling study with the capsular polysaccharides of *Streptococcus agalactiae* serotype III and *Streptococcus pneumoniae* serotype 14, leading to a conformational rationale for the antigenic epitopes identified for these polysaccharides. Based on their discovery they suggest a strategy for bacterial evasion of the host immune system by infection with these bacteria.

Chitosan-based films loaded with chitosan microparticles, that contain a bioactive peptide with antihypertensive properties, have been developed by Pintado and coworkers, consisting of an innovative approach to increase peptide efficiency and bioavailability.

McReynolds and coworkers established a new microwave-assisted oxime-based chemoselective methodology to prepare trivalent glycoclusters. The reaction is completed in 30 min, with the additional advantage of using unprotected sugars, and may be a step forward for the synthesis of more complex glycoconjugates and glycoclusters, multivalent molecules relevant for a number of biomedical uses.

Iminosugars are among the most relevant groups of glycomimetics for therapeutic applications. Among their variety of biological properties, their ability to mimic the transition state species in glycosidase catalysis and thus their propensity to inhibit these enzymes, which play a role in a variety of diseases, has led to some compounds which are used in clinics for the treatment of diabetes or Gaucher’s disease.

Two original articles in this special issue are devoted to the synthesis of new iminosugar derivatives and the evaluation of their glycosidase inhibitory properties.

Carvalho and coworkers investigated a small library of synthesized iminosugars differing in stereochemistry, ring size, and N-substitution and found two potent β-glucosidase inhibitors bearing d-*gluco* and l-*ido* configurations with six-membered and seven-membered ring iminosugars, in which the endocyclic amino group was derivatized with the hydroxyethyl group.

The contribution of Ramón Estevez and coworkers is based on the development of new synthetic routes to polyhydroxyoctahydroindoles, iminosugars with potential as pharmacological chaperones for lysosomal storage disorders, caused by mutations in the lysosomal β-galactosidase, and frequently related to misfolding. Resulting from abnormal metabolism of glycosphingolipids, glycogen, mucopolysaccharides or glycoproteins, they may generate neurodegenerative disorders, amongst others. The developed small molecules may act as ligands of the mutant enzyme, promoting the correct folding and preventing its degradation at the endoplasmic reticulum, a novel approach for disease treatment.

Alzheimer’s disease (AD) is also a neurodegenerative disorder, and drugs able to prevent disease progression are not yet available. Rauter and coworkers disclose the structure of C-glycosyl flavones as neuroprotective agents able to fully rescue human neuroblastoma cells from both H_2_O_2_ and Aβ1-42-induced cell death, a step forward to lead structures for further development against AD.

Another approach to treat AD patients is based on the cholinergic approach. Xavier and coworkers describe elegant syntheses of new purine and uracil isonucleosides embodying xylosyl or glucosyl groups, and the discovery of a potent and selective acetylcholinesterase inhibitor bearing a theobromine ring and an octyl chain linked to the glucosyl group.

Cell-surface glycans are recognized as therapeutic targets, as their composition changes in many diseases (e.g., in cancer). The review, authored by Rachel Hevey, covers approaches to develop glycomimetics that improve binding affinities and pharmacokinetic properties towards more drug-like compounds addressing therapies for carbohydrate-binding targets.

Hossain and Andreana revised the progress made in synthetic carbohydrate-based antitumor vaccines that improve immune responses by targeting specific antigens, in a beautiful work that also covers other developments in carbohydrate-based cancer treatments, including glycoconjugate prodrugs, glycosidase inhibitors, and early diagnosis.

We hope the readers enjoy this Special Issue and get inspired to unveil the secrets of life with carbohydrate sciences!

